# Detection of the c-myc oncogene product in testicular cancer.

**DOI:** 10.1038/bjc.1985.174

**Published:** 1985-08

**Authors:** K. Sikora, G. Evan, J. Stewart, J. V. Watson

## Abstract

**Images:**


*Br. J. Cancer* (1985), **52**, 171–176

### Detection of the c-*myc* oncogene product in testicular cancer

K. Sikora, G. Evan, J. Stewart & J.V. Watson

*Ludwig Institute for Cancer Research and MRC Clinical Oncology and Radiotherapeutics Unit, MRC Centre, Hills Road, Cambridge, UK.*

**Summary** A set of monoclonal antibodies was constructed by immunising mice with peptide fragments of the c-*myc* oncogene product. One such antibody, Myc 1-6E10 was shown to bind to a 62,000 dalton protein identifiable with the c-*myc* product ($p62^{c\text{-}myc}$). The antigen recognised was not destroyed by paraffin wax embedding. Myc 1-6E10 was used to characterise the distribution of $p62^{c\text{-}myc}$ in archival testicular tumour material. Normal testes expressed only small amounts of $p62^{c\text{-}myc}$. Seminomas showed increased nuclear and cytoplasmic staining. Undifferentiated teratoma showed little activity, whereas $p62^{c\text{-}myc}$ was abundant in the nuclei of differentiating epithelial structures, yolk sacs and embryoid bodiesl Only small amounts of $p62^{c\text{-}myc}$ were seen in the tumours of 5 patients who subsequently died from their disease.

The demonstration that human cancer cells may show abnormal expression of unique segments of DNA, called oncogenes, provides an exciting new avenue for clinical investigation. Over 25 of these genes have now been identified, molecularly cloned and sequenced (Krontiris, 1983; Hamlyn & Sikora, 1983). Changes in the coding or control regions of these genes have been implicated in the development of cancer (Cooper & Lane, 1984; Bishop, 1984). Several molecular mechanisms resulting in either the increased production of normal oncogene products or the development of aberrant proteins that subvert the normal growth control processes have now been uncovered (Der & Cooper, 1983; Stewart *et al.*, 1984*b*). These include gene amplification, translocation, mutation and rearrangement. Such changes have been documented in fresh tumour biopsies from patients as well as cultured cell lines (Rothberg *et al.*, 1984; Favera *et al.*, 1982). Using DNA hybridisation techniques, the N-*myc* gene has found to be amplified up to 100-fold in neuroblastoma (Schwab *et al.*, 1983) and retinoblastoma (Lee *et al.*, 1984). In one patient with chronic myelocytic leukaemia, the c-*myc* sequence was amplified 16-fold and rearranged within the genome during episodes of transformation (McCarthy *et al.*, 1984). Oncogene RNA transcripts have also been measured in fresh tumour material. K-*ras* and H-*ras* mRNA has been found elevated in colonic carcinoma, colonic polyps (Spandidos & Kerr, 1984) and breast cancer (Spandidos & Agnantis, 1984). Most interest has surrounded the *ras* and *myc* genes as considerable variation has been found in the quantity of their transcripts in clinical biopsies at the RNA level (Slamon *et al.*, 1984).

DNA and RNA hybridisation analysis is difficult to perform with many clinical samples. Low copy number genes and message cannot be detected with current methods. Hybridisation techniques cannot normally be applied to fixed embedded material stored in pathology departments. Furthermore they tell us nothing about the ultimate concentration and distribution in the cell of the gene product. The structure and function of these proteins are now under active investigation. At least one is related to a growth factor (c-*sis*) (Waterfield *et al.*, 1983) and another (c-*erb* B) to the internal component of the surface receptor for a growth factor (Downward *et al.*, 1984). The c-*myc* gene product is particularly intriguing with regard to human cancer. There is now evidence that this protein is associated with cell division and differentiation. The level of c-*myc* RNA increases when cells are stimulated into division (Kelly *et al.*, 1983). Both mRNA transcripts and the protein itself have unusually short half-lives of 20 to 30 min (Rabbitts *et al.*, 1985), a prerequisite for their putative cell cycle control function. Furthermore the protein appears to be associated with cell nuclei, a likely site for such control (Pauza & Evan, submitted for publication).

In order to examine the relevance of c-*myc* in clinical samples a set of mouse monoclonal antibodies against the c-*myc* protein was constructed (Evan *et al.*, 1985). The DNA sequence of the c-*myc* gene was used to determine the amino acid structure of the c-*myc* oncoprotein. Peptides of between 10 and 18 amino acids long were synthesized. The regions chosen for synthesis and immunisation were predicted to be exposed within the intact molecule by assessing the relative hydrophilicity of different parts of the sequence. Mice

Correspondence: K. Sikora
Received 1 March 1985; and in revised form, 1 May 1985.

© The Macmillan Press Ltd., 1985

were immunised to produce monoclonal antibodies (MCAs). Six MCAs were shown to bind to a 62,000 dalton protein identifiable with the c-*myc* product (p62$^{c\text{-}myc}$). These MCAs are currently being used to isolate and functionally characterise the c-*myc* protein.

In this report we demonstrate that one of these antibodies, Myc 1-6E10, can be used to detect p62$^{c\text{-}myc}$ protein in archival material from pathology laboratories. Because of the implication of this protein in growth and differentiation we chose to study tumour samples from patients with testicular cancer. Tumours of the testis arise predominantly from cells of the germ cell lineage (Ellis & Sikora, 1985). Malignant teratoma tissue often contains a wide range of differentiated cells identifiable by light microscopy in addition to undifferentiated and replicating stem cells. It therefore provides a biologically interesting system in which to examine the role of p62$^{c\text{-}myc}$ in differentiation as well as being of clinical interest.

**Figure 1** Schema for the production of MCA by peptide immunisation.

### Patients and methods

### *Monoclonal antibody*

Peptide synthesis, the immunisation protocol and screening procedures for deriving Myc 1-6E10 are described elsewhere (Evan *et al.*, 1985) and summarised in Figure 1. Hybridoma cells were grown in the ascites fluid of female BALB/c mice. The antibody was purified by octanoic acid precipitation followed by ammonium sulphate concentration. Purified antibody was adjusted to a concentration of $2\,mg\,ml^{-1}$ in PBS with 0.001% sodium azide, aliquoted and stored at $-20°C$. A control mouse immunoglobulin of the same sub-class (IgG1$\kappa$) was obtained from ascites fluid of the mouse myeloma line X-63 (Kohler & Milstein, 1975). It was similarly purified and adjusted to a concentration of $2\,mg\,ml^{-1}$.

### *Immunohistology*

Paraffin blocks containing surgical biopsies were obtained from the archives of the Department of Pathology. Thirty-two patients attending the Testicular Tumour Clinic at Addenbrooke's Hospital were studied. Sections ($5\,\mu m$) were cut and placed on standard microscope slides previously immersed in a 0.5% gelatin solution which also contained 250 mg chrome alum and 30 mg sodium azide $100\,ml^{-1}$ and air-dried overnight. An avidin-biotin technique was used to stain the sections (Vectorstain ABC Kit, Vector Labs). Sections were dewaxed and rehydrated using xylene and alcohol, incubated for 30 min with 0.5% hydrogen peroxide in methanol, washed and incubated for 20 min in diluted normal serum. Excess serum was blotted and the sections incubated for 60 min with $100\,\mu l$ Myc 1-6E10 diluted 1/500 in PBS, 1% BSA and 0.25% Triton X-100 (pH 7.3). Sections were washed and then incubated with a biotinylated rabbit anti-mouse immunoglobulin. After a further wash, sections were incubated for 45 min with the Vectorstain ABC reagent. A final incubation in diaminobenzidine (100 mg in 200 ml 0.5% hydrogen peroxide solution) was performed for 30 min and the sections washed with tap water and counterstained in Mayer's haemalum for 60 sec.

### Results

### *Specificity controls*

Most seminomas and teratomas showed considerable staining using Myc 1-6E10 when compared to normal testis. An irrelevant mouse monoclonal immunoglobulin (X63 IgG) of the same immunoglobulin subclass showed no binding. Staining by Myc 1-6E10 was blocked by the addition of 1 $\mu$g of the peptide used as the immunogen added to 100 $\mu$l of antibody prior to its addition to tumour sections (Table I).

**Table I** Specificity controls

| *First antibody* | *Seminoma* | *Teratoma* |
|---|---|---|
| Myc 1-6E10 | +++ | +++ |
| Myc 1-6E10+Peptide | – | – |
| Mouse X63 IgG1k | – | – |
| PBS | – | – |

*Normal testis*

Small amounts of $p62^{c\text{-}myc}$ were demonstrated in the cytoplasm and nuclei of the more peripheral spermatogonia of the normal seminiferous tubule (Figure 2a, b). The amount decreased towards the lumen of the tubules. It was also present in the cytoplasm of the interstitial cells of Leydig. There was no variation between different areas of the same testis.

*Seminoma*

Eleven patients with seminoma were studied. All showed increased $p62^{c\text{-}myc}$ staining predominantly located in cell nuclei but with some cytoplasmic increase. There was considerable variation between different areas of the same tumour and between different tumours (Figure 3). Infiltrating lymphocytes showed little or no staining.

*Malignant teratoma*

There was considerable variation in staining intensity between different parts of individual tumours in the 21 teratomas examined. Undifferentiated areas showed little $p62^{c\text{-}myc}$ activity, ofter less than the basal spermatogonia of normal testes. Areas of greatest intensity were clustered at sites of outgrowth of differentiating epithelial structures (Figure 4a b; Figure 5). Certain tumours, especially those with yolk sac differentiation, showed intense staining (Figures 6a, b). Little or no staining was seen in areas of trophoblastic differentiation (Table II).

No correlation of $p62^{c\text{-}myc}$ staining was observed between the stage of the disease or the preoperative serum level of human chorionic gonadotrophin. Five patients who subsequently died from their disease, and one who is currently undergoing chemotherapy for a recurrence, all had small amounts of the $p62^{c\text{-}myc}$ in their tumour.

**Figure 2** Transverse (a) and longitudinal sections (b) through a seminiferous tubule in a normal testis. $p62^{c\text{-}myc}$ present in small amounts in some spermatogonia ( $\times$ 400).

**Figure 3** Section of orchidectomy specimen from a patient with stage I testicular seminoma ( $\times$ 400).

Table II Immunohistological data

| *Tissue/tumour* | *p62$^{c\text{-}myc}$ distribution* |
|---|---|
| Normal testis | Small amounts of nuclear and cytoplasmic staining in spermatogonia at periphery of seminiferous tubules |
| Seminoma | Diffuse nuclear staining; little variation between tumours; no lymphocyte uptake |
| Malignant teratoma: | |
| Differentiated areas | Strong nuclear and cytoplasmic staining in developing epithelial areas; no staining in fully differentiated areas |
| Undifferentiated areas | Little staining |
| Trophoblastic | No staining |
| Yolk sac | Strong nuclear and cytoplasmic staining in lining cells; especially prominent in embryoid bodies |

**Figure 4** Sections through a specimen from a patient with malignant teratoma intermediate (MTI). p62$^{c\text{-}myc}$ is most prominent in the nuclei of differentiating cells at areas of outgrowth (a) ×200 (b) ×400.

**Figure 5** p62$^{c\text{-}myc}$ activity in nuclei and cytoplasm in differentiating areas of another malignant teratoma intermediate (×400).

**Figure 6** Embryoid body in patient with yolk sac malignant teratoma showing high p62$^{c\text{-}myc}$ activity in lining cells (a) ×200 (b) ×1000.

**Discussion**

The development of monoclonal antibodies to oncogene products provides essential reagents for characterising oncoprotein function and distribution in health and disease. The remarkable conservation of the DNA sequence of individual oncogenes across wide reaches of evolutionary time points to an essential role of their gene products in normal development. Testicular cancer thus provides a biologically as well as clinically relevant system for study.

The examination of c-*myc* mRNA in the developing human placenta indicates that the peak of *myc* transcription occurs at 4–5 weeks after conception (Pfeifer-Ohlsson *et al.*, 1984). Other oncogene RNA transcripts have been found to be elevated in differentiating systems. During liver regeneration the expression of H-*ras* and c-*myc* genes are increased (Goyette *et al.*, 1983) and the c-*fos* gene product is specifically elevated in developing bone (Muller *et al.*, 1982). In most cells the level of c-*myc* mRNA has been found to be low but increases with the rate of cell division (Pauza & Evan, submitted for publication). In the normal dividing germ cells of the mouse testis very few c-*myc* mRNA transcripts have been found. The drive to proliferate, at least for several divisions, may therefore come from other gene products. By partially purifying mouse testicular cells it has been demonstrated that c-*myc* expression was greatest in spermatogonia during the process of their differentiation into spermatocytes (Stewart *et al.*, 1984*a*). The histological studies described here suggest a similar pattern in normal human testis.

The control of cell division and differentiation are complex requiring the interaction of many different molecular mechanisms. The c-*myc* gene product is probably involved in both processes. Normal differentiation of germ cells requires the transient expression of high quantities of $p62^{c\text{-}myc}$. Malignant cells can also arise with several differentiation characteristics and containing various amounts of $p62^{c\text{-}myc}$. The most undifferentiated

**Figure 7** Model for c-*myc* expression during normal spermatogenesis and the development of germ cell tumours.

and therefore clinically aggressive teratomas contain low levels of this protein as measured by our antibody. Tumours can differentiate so expressing elevated levels, especially those with yolk sac elements. Fully differentiated tissue reverts to lower c-*myc* expression (Figure 7). This model accounts for the immunohistological observations in testicular cancer. Studying the expression of different gene products in histological material may result in greater precision of diagnosis and prognosis as well as opening new avenues for future therapy.

We would like to thank Professors J. Michael Bishop and Austin Gresham and Drs Sydney Brenner and Derek Wight for helpful discussion. J.S. holds a CRC Fellowship.

**References**

BISHOP, J.M. (1984). Cancer genes come of age. *Cell*, **32**, 1018.

COOPER, G.M. & LANE, M.A. (1984). Cellular transforming genes and oncogenesis. *Biochem. Biophys. Acta.*, **738**, 9.

DER, C.J. & COOPER, G.M. (1983). Altered gene products are associated with activation of cellular $ras^k$ genes in human lung and colon carcinomas. *Cell*, **32**, 201.

DOWNWARD, J., YARDEM, Y., MAYES, E. & 6 others. (1984). Close similarities of epidermal growth factor receptor and v-erb-B oncogene protein sequences. *Nature*, **307**, 521.

ELLIS, M. & SIKORA, K. (1985). Advances in the management of testicular cancer. In *Therapeutic Trials in Oncology*, p. 221. (Ed. Mathe). Bioscience: Geneva.

EVAN, G., LEWIS, G.K., RAMSAY, G. & BISHOP, J.M. (1985). Isolation of monoclonal antibodies specific for the human and mouse c-*myc* proto-oncogene products. *EMBO J.* (In press).

FAVERA, R.D., WONG-STAAL, F. & GALLO, R. (1982). *Onc* gene amplification in promyelocytic leukaemia cell line HL-60 and primary leukaemic cells of the same patient. *Nature*, **299**, 61.

GOYETTE, M., PETROPOULOS, C.J., SHANK, P.R. & FAUSTO, N. (1983). Expression of a cellular oncogene during liver regeneration. *Science*, **219**, 510.

HAMLYN, P. & SIKORA, K. (1983). Oncogenes. *Lancet*, **ii**, 326.

KELLY, K., COCHRAN, B.H., STILES, C.D. & LEDER, P. (1983). Cell specific regulation of the c-*myc* gene by lymphocyte mitogens and platelet derived growth factor. *Cell*, **35**, 603.

KOHLER, G. & MILSTEIN, C. (1975). Continuous cultures of fused cells provising antibodies of predefined specificity. *Nature*, **256**, 495.

KRONTIRIS, T.G. (1983). The emerging genetics of human cancer. *New Eng. J. Med.*, **309**, 404.

LEE, W.-W., MURPHEE, A.L. & BENEDICT, W.F. (1984). Expression and amplification of the N-*myc* gene in primary retinoblastoma. *Nature*, **309**, 458.

McCARTHY, D.M., RASSOOL, F.V., GOLDMAN, J.M., GRAHAM, S.V. & BIRNIE, G.D. (1984). Genomic alterations involving the c-*myc* proto-oncogene locus during the evolution of a case of chronic granulocytic leukaemia. *Lancet*, **ii**, 1362.

MULLER, R., SLAMON, D.J., TREMBLAY, J.M., CLINE, M. & VERNA, I.M. (1982). Differential expression of cellular oncogenes during pre- and post-natal development of the mouse. *Nature*, **299**, 640.

PFEIFER-OHLSSON, S., GOUSTIN, A.S., RYDNERT, J. & 4 others. (1984). Spatial and temporal pattern of cellular myc oncogene expression developing human placenta: Implications for embryonic cell proliferation. *Cell*, **38**, 585.

RABBITTS, P.H., LAMOND, A., WATSON, J.V. & 4 others. (1985). c-*myc* mRNA and protein metabolism in the cell cycle, *Cell*. (In press).

ROTHBERG, P.G., ERISMAN, M.D., DIEHL, R.E., ROVIATTI, U.G. & ASTRIN, S.M. (1984). Structure and expression of the oncogene c-*myc* in fresh tumour material from patients with haematopoietic malignancies. *Mol. Cell Biol.*, **4**, 1096.

SCHWAB, M., ALITALO, K., KLEMPNAUER, K.-H. & 6 others. (1983). Amplified with limited homology to *myc* cellular oncogene is shared by human neuroblastoma cell lines and a neuroblastoma tumour. *Nature*, **305**, 245.

SLAMON, D.J., DEKERNION, J.B., VERMA, I.M. & CLINE, M.J. (1984). Expression of cellular oncogenes in human malignancies. *Science*, **224**, 256.

SPANDIDOS, D.A. & AGNANTIS, N.J. (1984). Human malignant tumours of the breast, as compared to their respective normal tissue, have elevated expression of the Harvey *ras* oncogene. *Anticancer Research*, **4**, 269.

SPANDIDOS, D.A. & KERR, I.B. (1984). Elevated expression of the human *ras* oncogene family in premalignant and malignant tumours of the colorectum. *Br. J. Cancer*, **49**, 681.

STEWART, T.A., BELLVE, A. & LEDER, P. (1984*a*). Transcription and promoter usage of the *myc* gene in normal somatic and spermatogenic cells. *Science*, **226**, 707.

STEWART, T.A., PATTENGALE, P.K. & LEDER, P. (1984*b*). Spontaneous mammary adenocarcinomas in transgenic mice that carry and express MTV/myc fusion genes. *Cell*, **38**, 627.

WATERFIELD, M.D., SCRACE, G.T., WHITTLE, N. & 7 others. (1983). Platelet derived growth factor is structurally related to the putative transforming protein $p28^{sis}$ of simian sarcoma virus. *Nature*, **304**, 35.


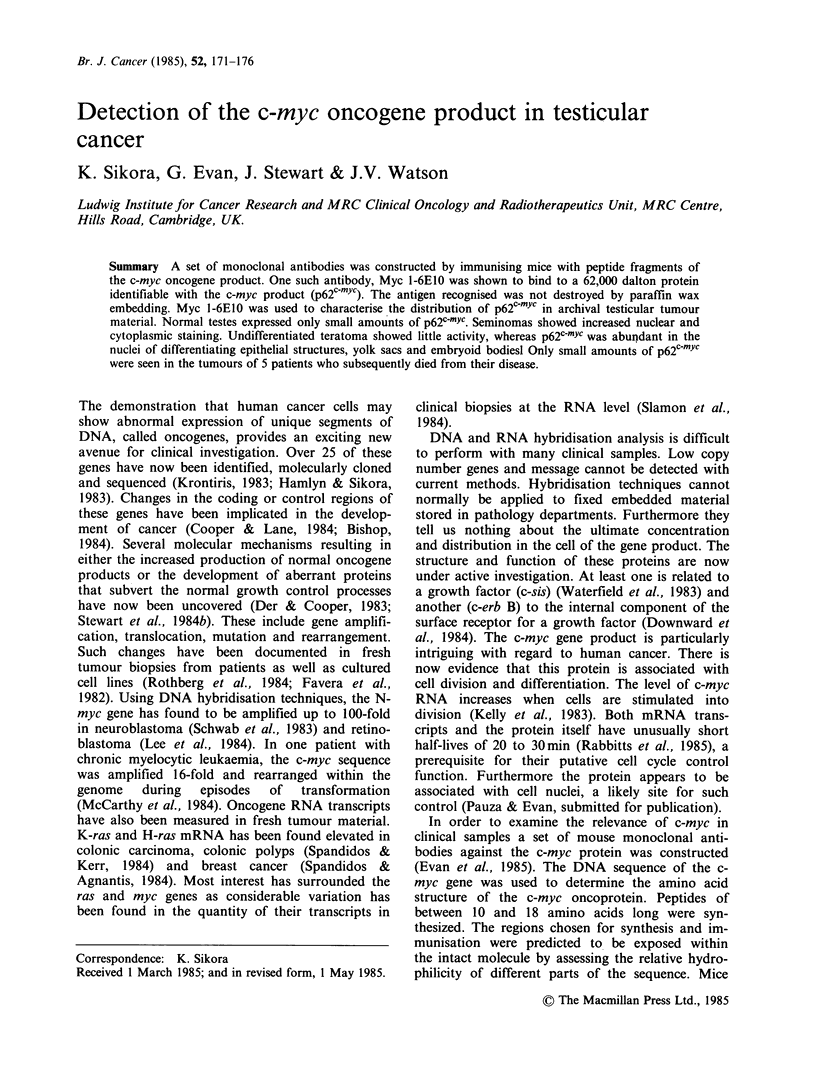

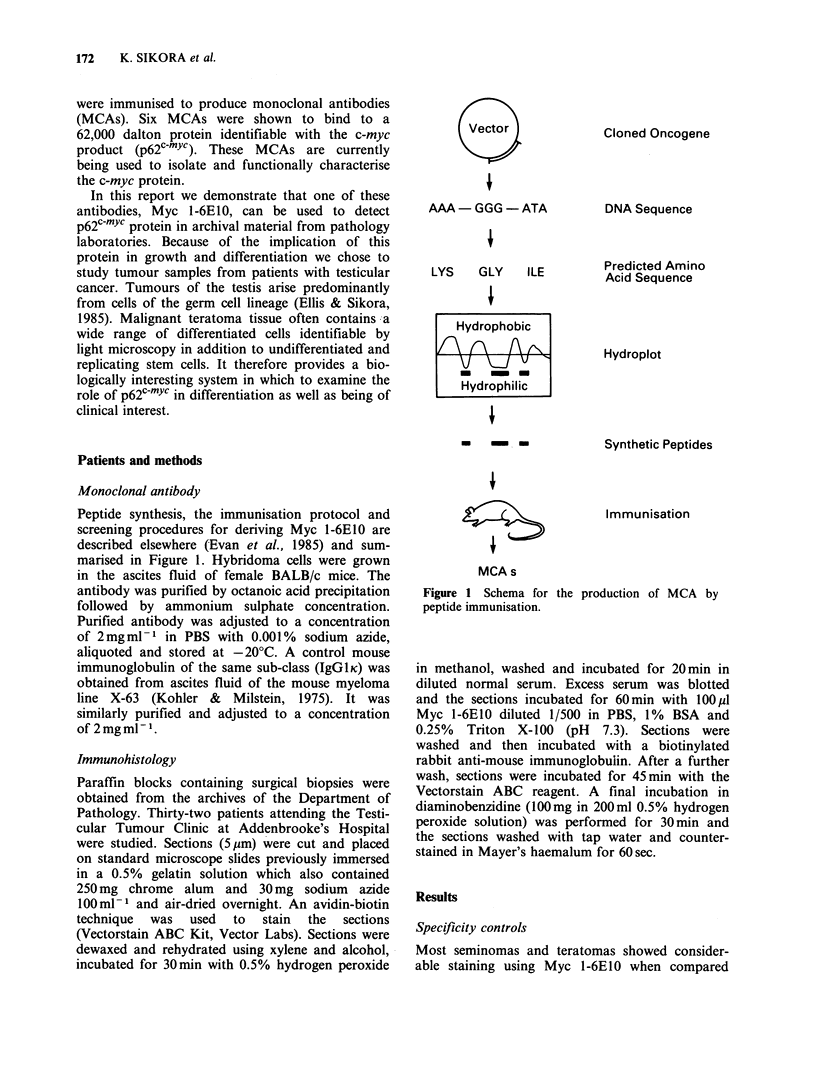

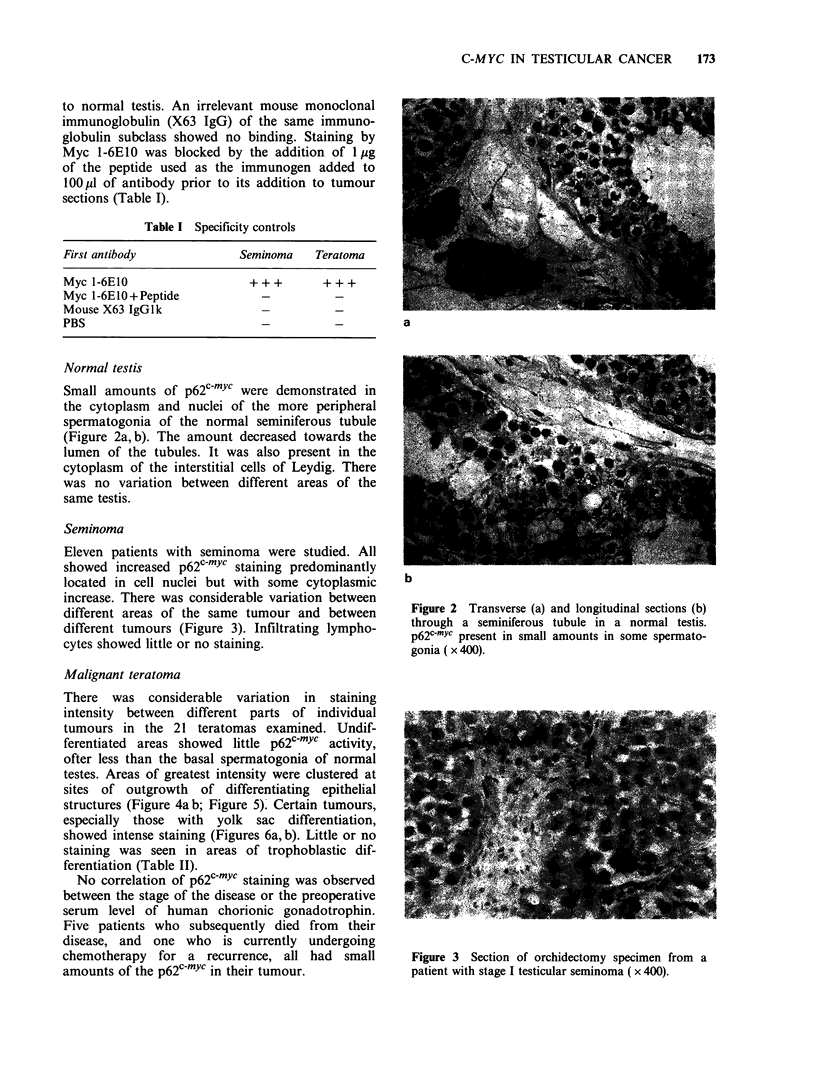

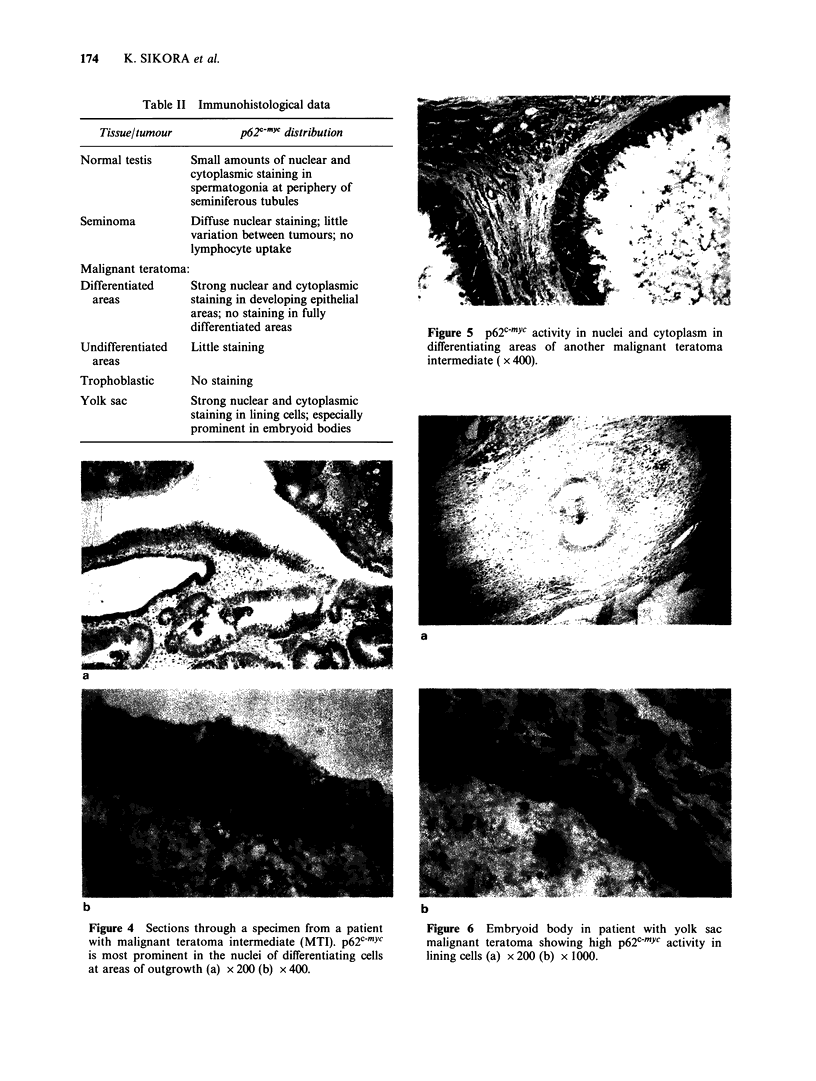

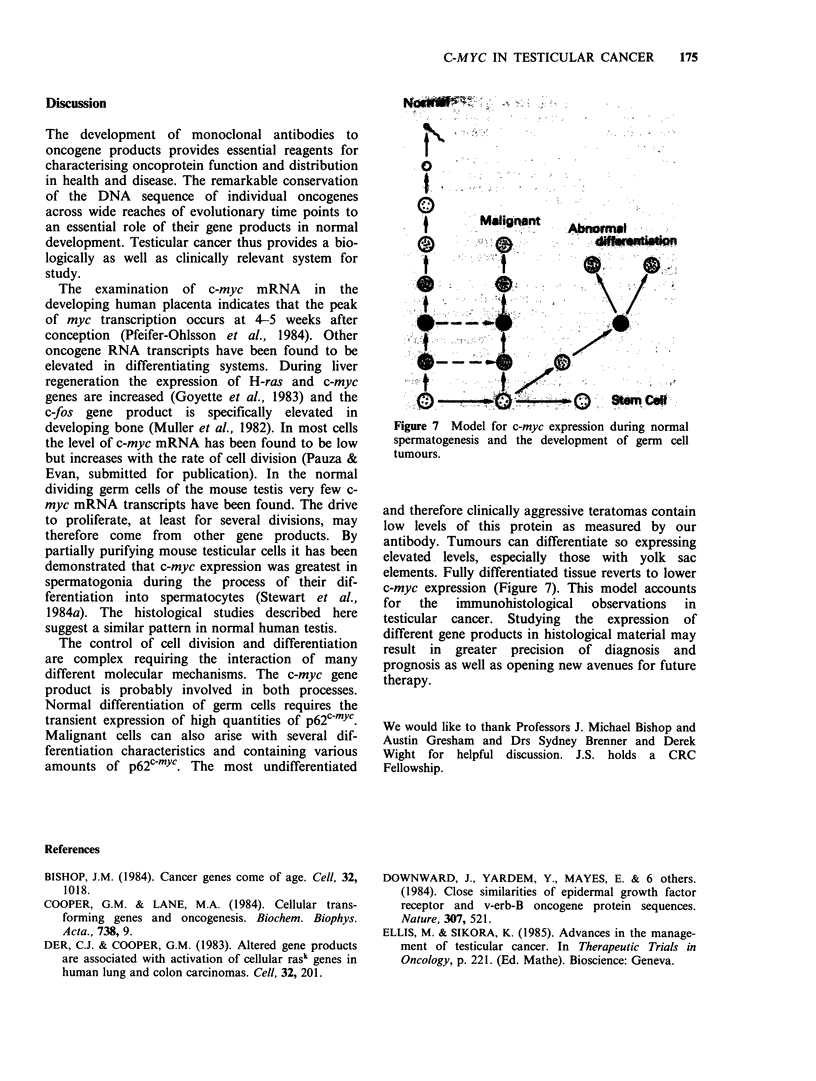

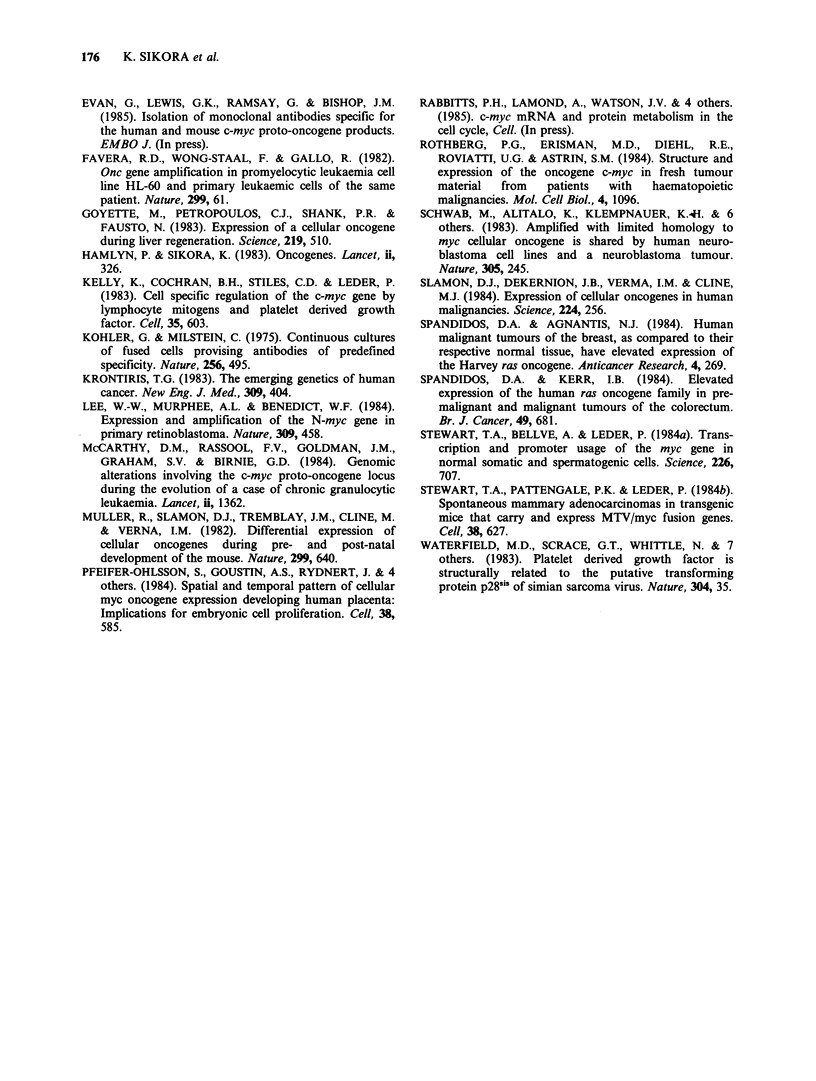


## References

[OCR_00422] Bishop J. M. (1983). Cancer genes come of age.. Cell.

[OCR_00426] Cooper G. M., Lane M. A. (1984). Cellular transforming genes and oncogenesis.. Biochim Biophys Acta.

[OCR_00455] Dalla-Favera R., Wong-Staal F., Gallo R. C. (1982). Onc gene amplification in promyelocytic leukaemia cell line HL-60 and primary leukaemic cells of the same patient.. Nature.

[OCR_00431] Der C. J., Cooper G. M. (1983). Altered gene products are associated with activation of cellular rasK genes in human lung and colon carcinomas.. Cell.

[OCR_00438] Downward J., Yarden Y., Mayes E., Scrace G., Totty N., Stockwell P., Ullrich A., Schlessinger J., Waterfield M. D. (1984). Close similarity of epidermal growth factor receptor and v-erb-B oncogene protein sequences.. Nature.

[OCR_00461] Goyette M., Petropoulos C. J., Shank P. R., Fausto N. (1983). Expression of a cellular oncogene during liver regeneration.. Science.

[OCR_00466] Hamlyn P., Sikora K. (1983). Oncogenes.. Lancet.

[OCR_00470] Kelly K., Cochran B. H., Stiles C. D., Leder P. (1983). Cell-specific regulation of the c-myc gene by lymphocyte mitogens and platelet-derived growth factor.. Cell.

[OCR_00481] Krontiris T. G. (1983). The emerging genetics of human cancer.. N Engl J Med.

[OCR_00476] Köhler G., Milstein C. (1975). Continuous cultures of fused cells secreting antibody of predefined specificity.. Nature.

[OCR_00485] Lee W. H., Murphree A. L., Benedict W. F. Expression and amplification of the N-myc gene in primary retinoblastoma.. Nature.

[OCR_00490] McCarthy D. M., Rassool F. V., Goldman J. M., Graham S. V., Birnie G. D. (1984). Genomic alterations involving the c-myc proto-oncogene locus during the evolution of a case of chronic granulocytic leukaemia.. Lancet.

[OCR_00497] Müller R., Slamon D. J., Tremblay J. M., Cline M. J., Verma I. M. (1982). Differential expression of cellular oncogenes during pre- and postnatal development of the mouse.. Nature.

[OCR_00503] Pfeifer-Ohlsson S., Goustin A. S., Rydnert J., Wahlström T., Bjersing L., Stehelin D., Ohlsson R. (1984). Spatial and temporal pattern of cellular myc oncogene expression in developing human placenta: implications for embryonic cell proliferation.. Cell.

[OCR_00515] Rothberg P. G., Erisman M. D., Diehl R. E., Rovigatti U. G., Astrin S. M. (1984). Structure and expression of the oncogene c-myc in fresh tumor material from patients with hematopoietic malignancies.. Mol Cell Biol.

[OCR_00522] Schwab M., Alitalo K., Klempnauer K. H., Varmus H. E., Bishop J. M., Gilbert F., Brodeur G., Goldstein M., Trent J. (1983). Amplified DNA with limited homology to myc cellular oncogene is shared by human neuroblastoma cell lines and a neuroblastoma tumour.. Nature.

[OCR_00529] Slamon D. J., deKernion J. B., Verma I. M., Cline M. J. (1984). Expression of cellular oncogenes in human malignancies.. Science.

[OCR_00534] Spandidos D. A., Agnantis N. J. (1984). Human malignant tumours of the breast, as compared to their respective normal tissue, have elevated expression of the Harvey ras oncogene.. Anticancer Res.

[OCR_00540] Spandidos D. A., Kerr I. B. (1984). Elevated expression of the human ras oncogene family in premalignant and malignant tumours of the colorectum.. Br J Cancer.

[OCR_00546] Stewart T. A., Bellvé A. R., Leder P. (1984). Transcription and promoter usage of the myc gene in normal somatic and spermatogenic cells.. Science.

[OCR_00552] Stewart T. A., Pattengale P. K., Leder P. (1984). Spontaneous mammary adenocarcinomas in transgenic mice that carry and express MTV/myc fusion genes.. Cell.

